# Effects of Nintedanib in an Animal Model of Liver Fibrosis

**DOI:** 10.1155/2020/3867198

**Published:** 2020-03-31

**Authors:** Lutz Wollin, Dieudonnée Togbe, Bernhard Ryffel

**Affiliations:** ^1^Immunology and Respiratory, Boehringer Ingelheim Pharma GmbH & Co. KG, Biberach 88397, Germany; ^2^Research and Development Department, Artimmune, Orléans 45100, France; ^3^Molecular Immunology, INEM, UMR7355, CNRS and University Orléans, Orléans 45071, France

## Abstract

Systemic sclerosis can affect multiple internal organs, including the liver and lungs. Nintedanib, an antifibrotic approved for treatment of interstitial lung disease associated with systemic sclerosis, may have activity outside of the lungs. This study explored the effect of preventive and therapeutic nintedanib treatment in a 3-week carbon tetrachloride (CCL_4_)-induced (500 mg/kg/day twice weekly for 3 weeks) model of hepatic inflammation and fibrosis in C57Bl/6 mice (aged 8 weeks, *n* = 5 per group). Mice also received nintedanib (30 or 60 mg/kg/day) either each day for 21 days (preventive treatment) or from day 7 or day 14 (therapeutic treatment). Preventive nintedanib treatment at both doses significantly reduced CCL_4_-induced increases in myeloperoxidase (*p* < 0.01), hepatic collagen (*p* < 0.001), and interleukin (IL)-6 (*p* < 0.01) in the liver. Nintedanib also significantly reduced hepatic necrosis (*p* < 0.01 and *p* < 0.05), inflammation (*p* < 0.001 and *p* < 0.05), fibrosis (*p* < 0.001 and *p* < 0.05) and IL-1*β* (*p* < 0.05 and *p* < 0.001) at both 30 and 60 mg/kg/day, respectively. Therapeutic treatment with nintedanib at 30 and 60 mg/kg/day significantly reduced CCL_4_-induced serum alanine aminotransferase from day 7 (*p* < 0.05 and *p* < 0.001) and day 14 (*p* < 0.01 and *p* < 0.05), respectively. Increases in tissue inhibitor of metalloproteinase-1 were significantly reduced by nintedanib at 60 mg/kg/day from day 7 only (*p* < 0.001), and nintedanib completely blocked elevation of IL-6 and IL-1*β* levels regardless of dose or start of treatment (*p* < 0.05–*p* < 0.001). In both the preventive and therapeutic treatment schedules of the study, nintedanib treatment was beneficial in attenuating CCL_4_-induced pathology and reducing hepatic injury, inflammation, and fibrosis, demonstrating that nintedanib has antifibrotic and anti-inflammatory activity outside of the lungs.

## 1. Introduction

Nintedanib is a small-molecule tyrosine kinase inhibitor that targets the receptors of certain fibrosis-related kinases, including PDGF, FGF, VEGF, and transforming growth factor-beta (TGF*β*) [1, 2]. Nintedanib also inhibits tyrosine kinases of the Src family such as Src, Lck, and Lyn and CSF1R that are involved in inflammation, proliferation, and immunological activation [2].

Systemic sclerosis (SSc) is a chronic disease characterised by endothelial dysfunction, resulting in alterations of the microvasculature, immune dysregulation, fibroblast dysfunction, and subsequent fibrosis [3]. Due to the heterogeneity of the disease, SSc remains a major clinical challenge for both physicians and patients [4]. The clinical course is variable, but SSc can affect multiple internal organs, particularly early in the disease, including the lungs, liver, kidneys, and heart [5, 6].

Nintedanib is an antifibrotic treatment currently approved in the United States, Canada, and Japan to slow the rate of decline in pulmonary function in adults with interstitial lung disease (ILD) associated with SSc (SSc-ILD) and in many countries for idiopathic pulmonary fibrosis (IPF). The INPULSIS [7] and TOMORROW [8] trials demonstrated the beneficial effects of nintedanib in patients with IPF. A pooled analysis of data from both trials showed that nintedanib was associated with a reduction in the annual rate of decline in forced vital capacity (FVC), fewer acute exacerbations, and the preservation of health-related quality of life [9].

Although the clinical course of SSc-ILD can vary, many patients experience disease progression with a decline in FVC associated with death [10, 11]. In the SENSCIS trial, nintedanib reduced the annual rate of decline in FVC compared with placebo [12]. The INBUILD trial evaluated nintedanib in patients with ILDs that have a progressive fibrosing phenotype; a subset of patients in the study have ILD associated with a connective tissue disease (CTD), e.g., rheumatoid arthritis (RA). Nintedanib reduced the annual rate of decline in FVC in the overall population, as well as in subgroups of patients with usual interstitial pneumonia-like or other fibrotic patterns [13]. Nintedanib has been well tolerated, with an acceptable safety profile and with diarrhoea the most common adverse event [7, 8, 12].

In addition to being associated with pulmonary manifestations, CTDs may also have an effect on the liver, typically as biochemical changes with a cholestatic or hepatocellular pattern. In rare cases, patients with CTDs may develop progressive liver disease, including liver fibrosis [14]. Given that multiple organs can be affected in patients with CTD, as well as the known antifibrotic and anti-inflammatory action of nintedanib, we planned to explore what effect nintedanib might have on liver injury.

Hepatic injury by carbon tetrachloride (CCL_4_) or ethanol causes inflammation, steatosis, and fibrosis that is toll-like receptor (TLR)4 and TGF*β*-signalling dependent [1] and models human liver cirrhosis. Nintedanib, which reduces TGF*β* and inflammatory mediators, may therefore have antifibrotic efficacy outside its known activity in the lungs. A previous preclinical acute study has shown antifibrotic effects of nintedanib in a 4-day CCL_4_ model of liver injury and fibrosis [15].

This study aimed to investigate whether nintedanib has antifibrotic activity outside of the lungs. The current study was undertaken to explore the effect of preventive and therapeutic nintedanib treatment in a 3-week CCL_4_-induced animal model of liver inflammation and fibrosis.

## 2. Materials and Methods

### 2.1. CCL_4_-Induced Model of Liver Fibrosis

We used a CCL_4_-induced model of hepatic inflammation and fibrosis in C57Bl/6 mice (Janvier, Le Genest-Saint-Isle, France). Mice were aged 8 weeks and caged in groups of five with *ad libitum* access to food and water. Animals received intraperitoneal CCL_4_ (Sigma-Aldrich, St. Louis, Missouri, United States) at 500 mg/kg/day twice weekly for 3 weeks to induce hepatic injury, inflammation, and fibrosis. Hepatic fibrosis was analysed at day 21. This study was approved by the regional ethics committee (CL2007-021).

### 2.2. Study Design

The study included two parts: a preventive and a therapeutic protocol. In the preventive phase, nintedanib treatment started simultaneously with CCL_4_ injections from day 0 to evaluate the effect of nintedanib in preventing hepatic injury. In the therapeutic phase, drug treatment was started on day 7 or day 14 of CCL_4_ injections to evaluate the effect of nintedanib in treating already established hepatic injury and fibrosis.

### 2.3. Treatment Protocols

**Figure 1:**
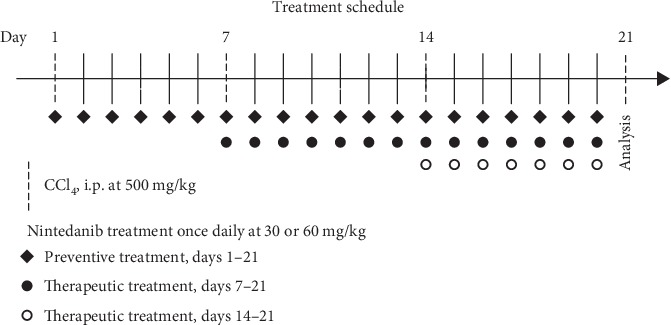
Treatment schedule in a mouse model of liver fibrosis. During the preventive phase, drug treatment started simultaneously with CCL_4_ injections from day 0. In the therapeutic phase, drug treatment was administered on day 7 or day 14 of CCL_4_ injections. Mice received nintedanib 30 or 60 mg/kg/day once daily. CCL_4_: carbon tetrachloride; i.p.: intraperitoneal.

Nintedanib 30 and 60 mg/kg/day (provided by Boehringer Ingelheim Pharma GmbH & Co. KG) was administered once daily by oral gavage. Administration volume was 10 mL/kg body weight. In the preventive protocol (Figure 1), there were four treatment groups (*n* = 10 per group): control (corn oil); CCL_4_ and oral vehicle; CCL_4_ and nintedanib 30 mg/kg/day; and CCL_4_ and nintedanib 60 mg/kg/day. In the therapeutic protocol, there were six treatment groups (*n* = 10 per group): control (corn oil); CCL_4_ and oral vehicle; CCL_4_ and nintedanib at 30 mg/kg/day starting at day 7; CCL_4_ and nintedanib 60 mg/kg/day starting at day 7; CCL_4_ and nintedanib at 30 mg/kg/day starting at day 14; and CCL_4_ and nintedanib 60 mg/kg/day starting at day 14.

### 2.4. Analyses

Body weight and clinical signs were recorded daily. All other analyses were conducted on day 21. Mice were euthanised at day 21 of the study, and the macroscopic analysis of the liver and liver weight changes was recorded postmortem. Alanine aminotransferase (ALT) and aspartate aminotransferase (AST) were determined in serum. Left liver lobes were homogenised to determine myeloperoxidase (MPO) activity, total liver collagen concentration by Sircol™ assay, interleukin (IL)-1*β*, IL-6, tumour necrosis factor-alpha (TNF*α*), and TGF*β* by enzyme-linked immunosorbent assay. Right liver lobes were fixed for histology (haematoxylin and eosin and Sirius Red F 3B staining) for semiquantitative assessment of liver necrosis, inflammatory cell infiltration, and fibrosis on a scale of 0–5 (0 = least severe and 5 = most severe). Details of the methods can be found in Seki et al. [1] and Lisbonne et al. [16].

All data are presented as mean ± standard error of the mean. Statistical differences between groups were analysed by one-way analysis of variance with subsequent Dunnett's multiple comparison test for all parametric data and Kruskal–Wallis test for nonparametric data (GraphPad Prism 5.04). Statistical significance was accepted at *p* < 0.05. All groups were tested against CCL_4_-treated positive control animals.

## 3. Results

### 3.1. Preventive Treatment

**Figure 2:**
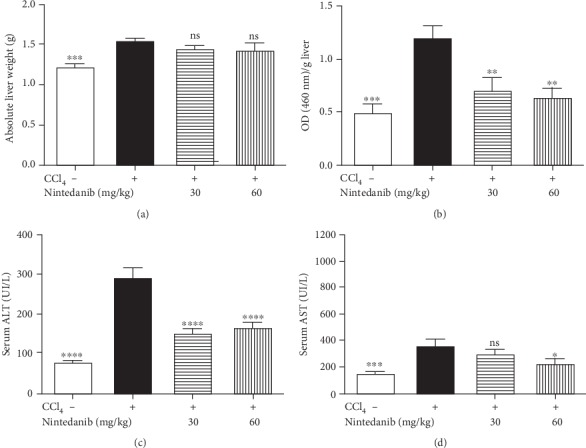
Effect of nintedanib on (a) liver weight and concentration in liver tissue of (b) myeloperoxidase (optical density), (c) serum alanine aminotransferase, and (d) serum aspartate aminotransferase during the preventive phase. Data are presented as mean ± standard error of the mean; *n* = 5 per group. Statistical significance is shown as: ^∗^*p* < 0.05, ^∗∗^*p* < 0.01, ^∗∗∗^*p* < 0.001, ^∗∗∗∗^*p* < 0.0001. ALT: alanine aminotransferase; ASP: aspartate aminotransferase; CCL_4_: carbon tetrachloride; ns: not significant.

Nintedanib treatment resulted in a dose-dependent weak trend towards reduced body weight (< 5% at 60 mg/kg/day) compared with control or CCL_4_-only treated animals, with no statistical difference at day 21 (data not shown). Nintedanib had no effect on CCL_4_-induced liver weight gain (Figure 2(a)), and no other alterations of major organs were observed. Neutrophil recruitment into the liver was assessed by MPO activity determined in liver homogenate (Figure 2(b)). Nintedanib treatment with either 30 or 60 mg/kg/day significantly reduced MPO activity (*p* < 0.01). In mice treated with nintedanib, there was a significant reduction of CCL_4_-induced increase in ALT at 30 and 60 mg/kg/day (*p* < 0.0001) and AST serum levels at 60 mg/kg/day (*p* < 0.05) (Figures 2(c) and 2(d)).

**Figure 3:**
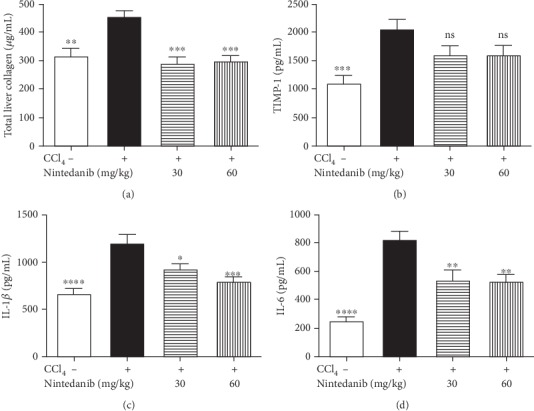
Effect of nintedanib on (a) total liver collagen and concentration in liver tissue of (b) TIMP-1, (c) IL-1*β*, and (d) IL-6 during the preventive phase. Data are presented as mean ± standard error of the mean; *n* = 5 per group. Statistical significance is shown as ^∗^*p* < 0.05, ^∗∗^*p* < 0.01, ^∗∗∗^*p* < 0.001, ^∗∗∗∗^*p* < 0.0001. CCL_4_: carbon tetrachloride; IL-1*β*: interleukin-1*β*; IL-6: interleukin-6; ns: not significant; TIMP-1: tissue inhibitor of metalloproteinase.

**Figure 4:**
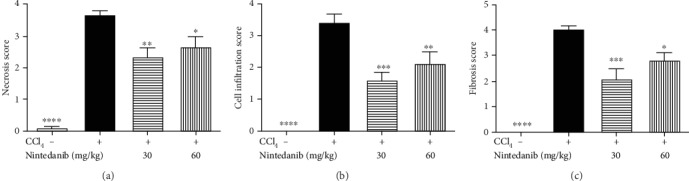
Effect of nintedanib on (a) liver necrosis, (b) liver inflammatory cell infiltration, and (c) liver fibrosis score during the preventive phase. Data are presented as mean ± standard error of the mean; *n* = 5 per group. Statistical significance is shown as ^∗^*p* < 0.05, ^∗∗^*p* < 0.01, ^∗∗∗^*p* < 0.001, ^∗∗∗∗^*p* < 0.0001. CCL_4_: carbon tetrachloride; ns: not significant.

**Figure 5:**
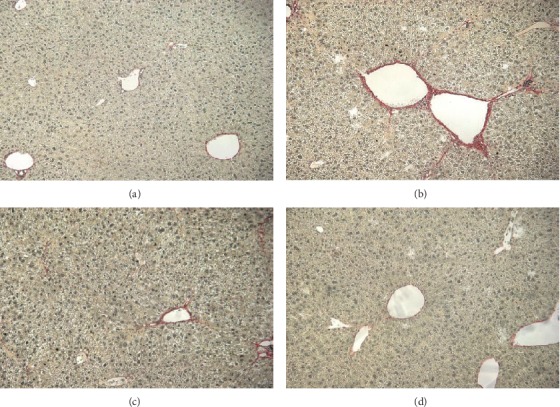
Representative histology slides stained with Sirius Red at 10-fold magnification during the preventive phase of (a) control without CCL_4_ stimulation, (b) control with CCL_4_ stimulation, (c) CCL_4_ and nintedanib at 30 mg/kg/day, (d) CCL_4_ and nintedanib 60 mg/kg/day. CCL_4_: carbon tetrachloride.

Nintedanib at both doses blocked CCL_4_-induced increase in hepatic collagen (*p* < 0.001) (Figure 3(a)). Nintedanib reduced tissue inhibitor of metalloproteinase (TIMP)-1, IL-1*β*, and IL-6 in the liver (Figures 3(b)–3(d)). This reduction was significant at both doses for IL-6 (*p* < 0.01) and for IL-1*β* (30 mg/kg/day; *p* < 0.05 and 60 mg/kg/day; *p* < 0.001). TNF*α*, TGF*β*, and C-X-C motive ligand-1 (KC) levels were not affected by nintedanib administration (data not shown). Nintedanib significantly reduced liver necrosis (*p* < 0.01 and *p* < 0.05), infiltration of inflammatory cells (*p* < 0.001 and *p* < 0.01), and fibrosis (*p* < 0.001 and *p* < 0.05) at both 30 and 60 mg/kg/day, respectively, as assessed by semiquantitative scoring of histology samples (Figures 4 and 5).

### 3.2. Therapeutic Treatment

**Figure 6:**
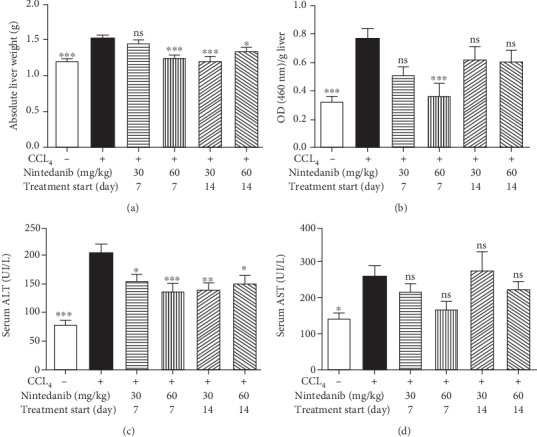
Effect of nintedanib on (a) liver weight and concentration in liver tissue of (b) myeloperoxidase, (c) alanine aminotransferase, and (d) aspartate aminotransferase during the preventive phase. Data are presented as mean ± standard error of the mean; *n* = 5 per group. Statistical significance is shown as ^∗^*p* < 0.05, ^∗∗^*p* < 0.01, ^∗∗∗^*p* < 0.001. ALT: alanine aminotransferase; ASP: aspartate aminotransferase; CCL_4_: carbon tetrachloride; ns: not significant.

Nintedanib treatment resulted in a weak trend towards reduced body weight (< 5% at 60 mg/kg/day) compared with control or CCL_4_-only treated animals from both days 7 and 14 (data not shown). Macroscopic examination of the major organs at necropsy on day 21 did not reveal any major organ alterations. Nintedanib significantly reduced CCL_4_-induced liver weight gain at both 30 mg/kg/day (from day 14; *p* < 0.001) and 60 mg/kg/day (from day 7 or 14; *p* < 0.001 and *p* < 0.05, respectively) (Figure 6(a)). There was a dose-dependent decrease in MPO if treatment started on day 7 only, reaching significance with 60 mg/kg/day (*p* < 0.001) (Figure 6(b)). ALT was significantly reduced following treatment with both doses of nintedanib, with treatment starting on either day 7 (*p* < 0.05 and *p* < 0.001) or 14 (*p* < 0.01 and *p* < 0.05) (Figure 6(c)). There was a nonsignificant trend towards dose-dependent reduction in AST (Figure 6(d)).

**Figure 7:**
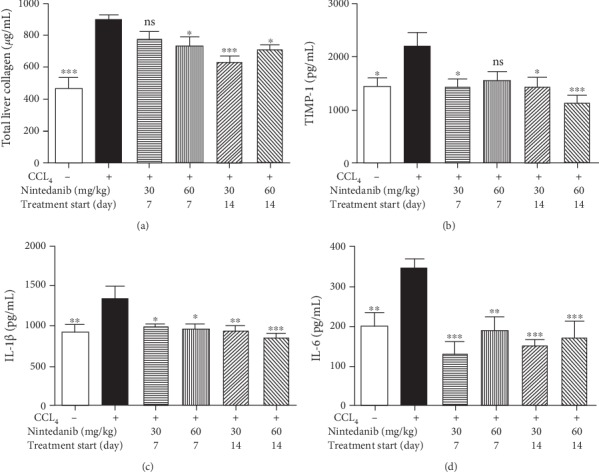
Effect of nintedanib on (a) total liver collagen and concentration in liver tissue of (b) TIMP-1, (c) IL-1*β*, and (d) IL-6 during the therapeutic phase. Data are presented as mean ± standard error of the mean; *n* = 5 per group. Statistical significance is shown as ^∗^*p* < 0.05, ^∗∗^*p* < 0.01, ^∗∗∗^*p* < 0.001. CCL_4_: carbon tetrachloride; IL-1*β*: interleukin-1*β*; IL-6: interleukin-6; ns: not significant; TIMP-1: tissue inhibitor of metalloproteinase.

**Figure 8:**
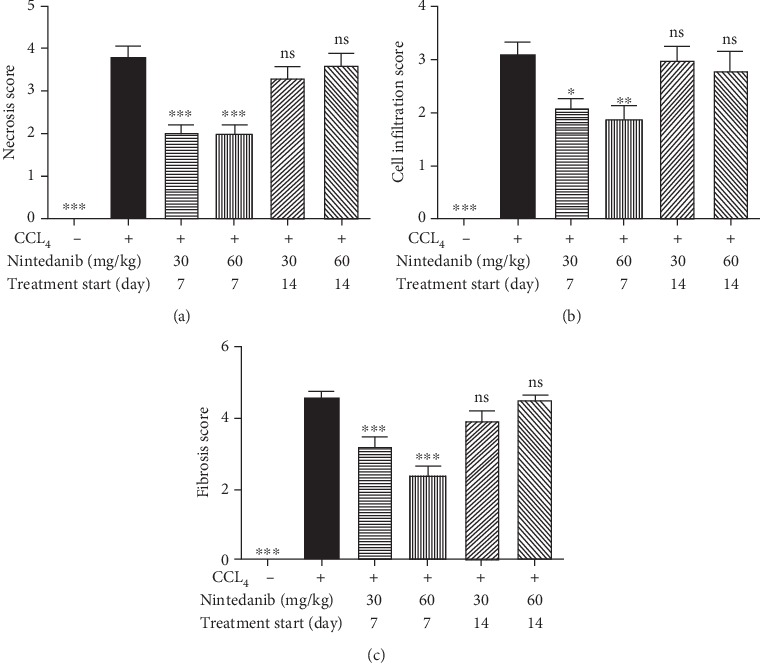
Effect of nintedanib on (a) liver necrosis, (b) liver inflammatory cell infiltration, and (c) liver fibrosis score during the therapeutic phase. Data are presented as mean ± standard error of the mean; *n* = 5 per group. Statistical significance is shown as ^∗^*p* < 0.05, ^∗∗^*p* < 0.01, ^∗∗∗^*p* < 0.001. CCL_4_: carbon tetrachloride; ns: not significant.

Nintedanib significantly reduced the total liver collagen concentration at 30 mg/kg/day (*p* < 0.001, from day 14) and at 60 mg/kg/day (from day 7 or 14, *p* < 0.05) (Figure 7(a)). Increases in TIMP-1 were significantly reduced by nintedanib 30 mg/kg/day from days 7 and 14 (*p* < 0.05), and at 60 mg/kg/day from day 14 only (*p* < 0.001) (Figure 7(b)). Nintedanib completely blocked elevation of IL-6 and IL-1*β* levels regardless of dose or start of treatment (*p* < 0.05–*p* < 0.001) (Figures 7(c) and 7(d)). TNF*α*, TGF*β*, and KC levels were not affected by nintedanib administration (data not shown). Nintedanib treatment at both doses significantly reduced liver necrosis, inflammation, and fibrosis, as assessed by semiquantitative histology, from day 7 only (Figures 8(a)–8(c)). The reduction in liver fibrosis appeared to be greater at 60 mg/kg/day than at 30 mg/kg/day (Figure 8(c)).

## 4. Discussion

In both the preventive and therapeutic treatment schedules of the study, nintedanib treatment attenuated CCL_4_-induced pathology and reduced hepatic injury, inflammation, and fibrosis.

In the preventive setting, there was a trend towards reduced ALT and AST in serum. Nintedanib treatment diminished neutrophil recruitment and reduced biochemical and inflammatory parameters suggestive of a beneficial effect on liver fibrosis. Both doses of nintedanib had similar preventive activities on most parameters explored but with no dose dependency.

Nintedanib treatment starting at day 7 was, in general, more effective than treatment starting at day 14; liver necrosis, inflammatory cell infiltration, and liver fibrosis, when assessed histologically, were only significantly reduced if treatment was started on day 7. This may have been because the pathology had already progressed too far or because treatment duration was too short to be effective when starting at day 14 (only 7 days of treatment). However, this study was not designed to describe statistically significant differences between treatments started at different time points.

ALT/AST results were different from those seen in clinical trials, where elevated ALT/AST levels at least three times the upper limit of the normal range were observed in 4.9–13.0% of patients with lung fibrosis treated with nintedanib compared with placebo (up to 3.6%), depending on the study [7, 8, 12, 13]. In our mouse model, nintedanib did not increase ALT or AST serum levels. It may be that only patients with already impaired liver function before the start of clinical trials are prone to the increases in ALT and AST following nintedanib treatment.

Similar to our findings, another preclinical model of CCL_4_-induced liver fibrosis found that nintedanib significantly attenuated collagen accumulation and hepatic stellate cell activation, inhibiting intrahepatic inflammation and angiogenesis [15]. In a choline-deficient, L-amino acid-defined, high-fat diet-fed mouse model of nonalcoholic steatohepatitis, nintedanib also showed anti-inflammatory and antifibrotic effects [17].

Biochemical changes in the liver are common in patients with CTD. In rare cases, patients with CTDs may develop progressive liver disease, including liver fibrosis [14]. Liver involvement of this type is typically attributed to (coexisting) primary liver diseases (e.g., fatty liver disease, viral hepatitis, primary biliary cirrhosis, autoimmune hepatitis, and drug-related liver toxicity), rather than the CTD itself. Drug-induced liver injury is frequent with certain CTDs, particularly with the use of methotrexate in patients with RA, and may lead to progressive fibrosis and cirrhosis [18]. The results of the current study highlight the potential of nintedanib to reduce liver necrosis, inflammation, and fibrosis. As such, using nintedanib for the treatment of ILD in patients with CTDs, including SSc-ILD, may have antifibrotic effects in organs other than the lungs.

Nintedanib reduced and blocked CCL_4_-induced elevated hepatic IL-6 in both the preventive and therapeutic phases of the study. Similar results were obtained in a mouse model of silica-induced lung fibrosis, where nintedanib reduced the IL-6 concentration in lung tissue [19]. Anti-IL-6 antibody therapy is used to treat RA-ILD, although evidence is controversial, with some studies showing beneficial effects and others finding ILD occurrence or exacerbation following tocilizumab treatment [20, 21]. Interestingly, imatinib mesylate, another kinase inhibitor that has shown antifibrotic effects in the liver in preclinical studies, has been shown to increase serum IL-6 levels in conjunction with liver regeneration [22]. The current study shows a potential systemic effect of nintedanib on IL-6, indicating that further investigation of nintedanib in RA-ILD may be warranted.

However, there are limitations with this study, including those normally associated with animal models of disease. The CCL_4_ model is also more a model of acute liver injury leading to fibrosis and does not directly correspond to the clinical situation where patients normally present with chronic fibrosis [15]. However, it provides a good indication (along with other animal models of fibrosis discussed above) that nintedanib has antifibrotic activity outside of the lungs.

## 5. Conclusions

In conclusion, nintedanib demonstrated antifibrotic and anti-inflammatory activity, showing therapeutic potential in this experimental mouse model of CCL_4_-induced hepatic fibrosis.

## Data Availability

To ensure independent interpretation of clinical study results, Boehringer Ingelheim grants all external authors access to all relevant material, including participant-level clinical study data, and relevant material as needed by them to fulfil their role and obligations as authors under the International Committee of Medical Journal Editors criteria. Furthermore, clinical study documents (e.g. study report, study protocol, statistical analysis plan) and participant clinical study data are available to be shared after publication of the primary manuscript in a peer-reviewed journal, and if regulatory activities are complete and other criteria are met per the BI Policy on Transparency and Publication of Clinical Study Data: https://trials.boehringer-ingelheim.com/transparency_policy.html. Prior to providing access, documents will be examined, and, if necessary, redacted and the data will be de-identified, to protect the personal data of study participants and personnel, and to respect the boundaries of the informed consent of the study participants. Clinical Study Reports and Related Clinical Documents can be requested via this link: https://trials.boehringer-ingelheim.com/trial_results/clinical_submission_documents.html. All such requests will be governed by a Document Sharing Agreement. Bona fide, qualified scientific and medical researchers may request access to de-identified, analysable participant clinical study data with corresponding documentation describing the structure and content of the data sets. Upon approval, and governed by a Data Sharing Agreement, data are shared in a secured data-access system for a limited period of 1 year, which may be extended upon request. Researchers should use https://clinicalstudydatarequest.com to request access to study data.
